# Speech emotion recognition based on improved masking EMD and convolutional recurrent neural network

**DOI:** 10.3389/fpsyg.2022.1075624

**Published:** 2023-01-09

**Authors:** Congshan Sun, Haifeng Li, Lin Ma

**Affiliations:** Faculty of Computing, Harbin Institute of Technology, Harbin, China

**Keywords:** speech emotion recognition, empirical mode decomposition, mode mixing, convolutional neural networks, bidirectional gated recurrent units

## Abstract

Speech emotion recognition (SER) is the key to human-computer emotion interaction. However, the nonlinear characteristics of speech emotion are variable, complex, and subtly changing. Therefore, accurate recognition of emotions from speech remains a challenge. Empirical mode decomposition (EMD), as an effective decomposition method for nonlinear non-stationary signals, has been successfully used to analyze emotional speech signals. However, the mode mixing problem of EMD affects the performance of EMD-based methods for SER. Various improved methods for EMD have been proposed to alleviate the mode mixing problem. These improved methods still suffer from the problems of mode mixing, residual noise, and long computation time, and their main parameters cannot be set adaptively. To overcome these problems, we propose a novel SER framework, named IMEMD-CRNN, based on the combination of an improved version of the masking signal-based EMD (IMEMD) and convolutional recurrent neural network (CRNN). First, IMEMD is proposed to decompose speech. IMEMD is a novel disturbance-assisted EMD method and can determine the parameters of masking signals to the nature of signals. Second, we extract the 43-dimensional time-frequency features that can characterize the emotion from the intrinsic mode functions (IMFs) obtained by IMEMD. Finally, we input these features into a CRNN network to recognize emotions. In the CRNN, 2D convolutional neural networks (CNN) layers are used to capture nonlinear local temporal and frequency information of the emotional speech. Bidirectional gated recurrent units (BiGRU) layers are used to learn the temporal context information further. Experiments on the publicly available TESS dataset and Emo-DB dataset demonstrate the effectiveness of our proposed IMEMD-CRNN framework. The TESS dataset consists of 2,800 utterances containing seven emotions recorded by two native English speakers. The Emo-DB dataset consists of 535 utterances containing seven emotions recorded by ten native German speakers. The proposed IMEMD-CRNN framework achieves a state-of-the-art overall accuracy of 100% for the TESS dataset over seven emotions and 93.54% for the Emo-DB dataset over seven emotions. The IMEMD alleviates the mode mixing and obtains IMFs with less noise and more physical meaning with significantly improved efficiency. Our IMEMD-CRNN framework significantly improves the performance of emotion recognition.

## 1. Introduction

Emotion is a kind of physiological and psychological state (Liu Z. et al., 2022). Physiological stimulation, subjective experience, and facial and behavioral expression all work together to form a complete emotional process (Nitsche et al., 2012; Lu et al., 2021). Basic emotional states comprise anger, disgust, fear, happiness, sadness, and surprise (Ekman and Friesen, 1971). The remaining emotions are combinations of these basic emotions, such as excitement, embarrassment, and contempt (Krishnan et al., 2021). Reliable analysis, recognition, understanding, and expression of emotions are significant for communicating and understanding information between humans and computers.

Attempts utilizing separate modalities have been made to recognize emotions (Aydın et al., 2018; Dominguez-Jimenez et al., 2020; Li et al., 2020a,b). Accumulating evidence have proved the efficiencies of EEG and other physiological signals (such as electrocardiograph, galvanic skin response, and respiration) in emotion recognition (Quan et al., 2021; Chen et al., 2022). In these experiments, physiological signals were simultaneously recorded while subjects were presented with diversified emotional stimulus materials (such as static pictures, facial expressions, video film clips, and acoustic music clips) that induced specific emotions, among which the parameters of these stimulus materials would also influence the intensity of induced emotions (Kılıç and Aydın, 2022). For emotion recognition, emotional features of EEG signals usually include power spectrum density (PSD), differential entropy (DE), rational asymmetry (RASM), differential entropy asymmetry (DASM), phase locking value (PLV), and phase lag index (PLI; Lu et al., 2021). For other physiological signals, some statistical features based on temporal or frequency-domain information are usually extracted for emotion recognition (Picard et al., 2001; Goshvarpour et al., 2017).

Speech is one of the most natural and intuitive ways of emotional communication, which contains rich emotions while conveying information (Li et al., 2020a). Speech emotion recognition (SER) is a computer simulation of human speech emotion perception and understanding, a key prerequisite for human-computer interaction. There are three main methods for emotional corpora collection: collecting natural speech from the real world (natural speech database), collecting audio recordings of subjects acting based on pre-decided affect-related scripts (actor-based speech database), and collecting corpora from the speaker by creating an artificial emotional situation (elicited emotional speech database; Basu et al., 2017). Emotional features of speech signals include prosody features, spectral features, and timbre features (Li et al., 2020b). The current SER is mainly supervised pattern recognition. Commonly used machine learning algorithms include k-nearest neighbor (KNN), support vector machine (SVM), linear discriminative analysis (LDA), Gaussian naive Bayes, and artificial neural network (ANN).

With the development of deep learning, SER based on deep neural networks (DNNs) has begun to attract attention. These methods train deep-learning models for speech emotion recognition by taking the original emotional speech or hand-crafted features as the inputs and have achieved fruitful results (Anvarjon et al., 2020). Sarma et al. (2018) identified emotions from raw speech signals using an interleaving time-delay neural network (TDNN) with unidirectional long short-term memory (LSTM) and time-restricted attention mechanisms (TDNN-LSTM-attention). The results outperformed previously reported results on the IEMOCAP dataset (Busso et al., 2008). Wang et al. (2021) proposed a novel end-to-end SER architecture that stacked multiple transformer layers and used log Mel-filterbank energy features as the input. This method outperformed prior methods by a relatively 20% improvement on the IEMOCAP dataset. Deschamps-Berger et al. (2021) presented an end-to-end temporal CNN-BiLSTM network and extracted the spectrogram by short-term Fourier transform (STFT) as the input of the network. This method was evaluated on the IEMOCAP and CEMO datasets and obtained good results. Kim and Saurous (2018) used two CNN layers for local and global convolution, two LSTM layers for sequence learning, and 20 features from eGeMAPs (containing rhythmic, spectral, and timbre features) as inputs to the model. On the Emo-DB dataset, an unweighted accuracy of 88.9% was achieved. Wang et al. (2022) extracted traditional hand-crafted features from GeMAPS and deep automatic features from the VGGish model. Then, they proposed a multi-feature fusion and Multi-lingual fusion speech emotion recognition algorithm based on the recurrent neural network (RNN) with an improved local attention mechanism. The speech emotion recognition accuracy is improved when the dataset is small. Hou et al. (2022) proposed a collective multi-view relation network (CMRN) based on bidirectional gate recurrent units (Bi-GRU) and the attention mechanism. In the CMRN, Mel-frequency cepstral coefficients (MFCCs), log Mel-frequency spectral coefficients (MFSCs), and prosody features are collected as multi-view representations. The proposed method performs better than the state-of-the-art methods on Emo-DB and IEMOCAP datasets.

For actual voice, automatic feature learning methods using deep networks can effectively learn the underlying patterns in the data. However, it is not easy to interpret the information obtained from these deep networks (Bhattacharjee et al., 2020). On the other hand, hand-crafted features used in deep-learning methods are mainly extracted based on the STFT. In practical applications, speech signals are non-stationary amplitude modulated-frequency modulated (AM-FM) signals with rich frequency components and temporal rhythm variations (Hsieh and Liu, 2019). The nonlinear features of speech emotion are variable, complex, and subtly changing (Kerkeni et al., 2019). However, limited by the fundamental uncertainty principle, the STFT cannot get good resolution in both time and frequency, and the non-linearity issue remains problematic (Kerkeni et al., 2019). Meanwhile, the STFT method requires pre-set basis functions and lacks adaptiveness in analyzing non-stationary speech (Yang et al., 2018). Therefore, reliable recognition of emotions from speech remains challenging.

More recently, empirical mode decomposition (EMD), a decomposition method for non-stationary AM-FM signals, has been used to analyze emotional speech signals. EMD adaptively decomposes a non-stationary signal into a finite number of intrinsic mode functions (IMFs) without losing the original properties of signals (Huang et al., 1998). IMFs have been shown to manifest the vocal tract structure and the glottal source information (Sharma et al., 2018; Karan et al., 2020). At the same time, experimental studies have shown that variations in the physiological properties of the vocal folds vary significantly across emotional patterns (Yao et al., 2020). Therefore, good results are obtained for speech emotion recognition based on EMD. Based on empirical mode decomposition (EMD) and Teager-Kaiser energy operator (TKEO), Kerkeni et al. (2019) extracted two new types of features. Combining these two feature sets with cepstral features, the unweighted accuracy using the support vector machine (SVM) on the Emo-DB dataset is 86.22%. Vieira et al. (2020) presented a novel Hilbert–Huang–Hurst coefficient (HHHC) feature based on the ensemble EMD (EEMD) to represent the emotional states. Experiments on different emotional datasets showed that HHHC led to significant classification improvements compared to the baseline acoustic features. Krishnan et al. (2021) extracted entropy features from principal IMFs based on EMD for recognizing emotions on the TESS dataset and the linear discriminant analysis (LDA) classifier presented a peak balanced accuracy of 93.3%. However, EMD and EEMD suffer from the mode mixing problem, which makes the physical meaning of IMF unclear (Rilling and Flandrin, 2008), thus reducing the performance of EMD-based methods for speech emotion recognition. Researchers have proposed several improvement methods for the mode mixing problem, such as the masking signal-based EMD (MSEMD; Deering and Kaiser, 2005), improved complete ensemble EMD with adaptive noise (ICEEMDAN; Colominas et al., 2014), uniform phase EMD (UPEMD; Wang et al., 2018), and robust EMD (REMD; Liu P. et al., 2022). Although these methods alleviate the modal aliasing problem to some extent, there are still problems in that the method parameters cannot be determined adaptively, there is residual noise in the IMFs, and the time complexity of the algorithm is high.

It is still challenging for computers to accurately capture emotional information in speech (Anvarjon et al., 2020). Therefore, this paper focuses on exploring and proposing an effective SER method to help computers develop advanced emotional intelligence. In this paper, we present a novel framework, named IMEMD-CRNN, to address the above challenges and improve speech-based emotion recognition performance.

The contributions of this work are three-fold: (i) We propose an improved version of the masking signal-based EMD (IMEMD). In the IMEMD, the parameters of masking signals are adaptively derived from the natures of the original signals. IMEMD obtains IMFs with less noise and more physical meaning with significantly improved efficiency. (ii) We use IMEMD to extract the timbre features proposed in our previous work (Li et al., 2020b) and Mel-frequency cepstral coefficients based on the reconstructed signal (SMFCC; Kerkeni et al., 2019) as the features used in the IMEMD-CRNN to characterize speech emotions. These are important speech emotion features (Guidi et al., 2019; Kerkeni et al., 2019). (iii) We feed the timbre features based on IMEMD into a convolutional recurrent neural network (CRNN) to recognize emotions. In the CRNN, we first use 2D CNN layers to capture nonlinear local temporal and frequency information of the emotional speech. Then, the outputs of the CNN module are fed to bidirectional gated recurrent units (BiGRU) layers to learn the temporal context information further. In the experimental part, we first demonstrated the advantages of IMEMD for decomposing non-stationary signals through the performance of the different improved algorithms for EMD in simulated and real speech emotion signals. Then experiments on two popular standard speech emotion datasets showed the significance and the robustness of our proposed IMEMD-CRNN framework for speech emotion recognition.

## 2. Materials and methods

**Figure 1:**
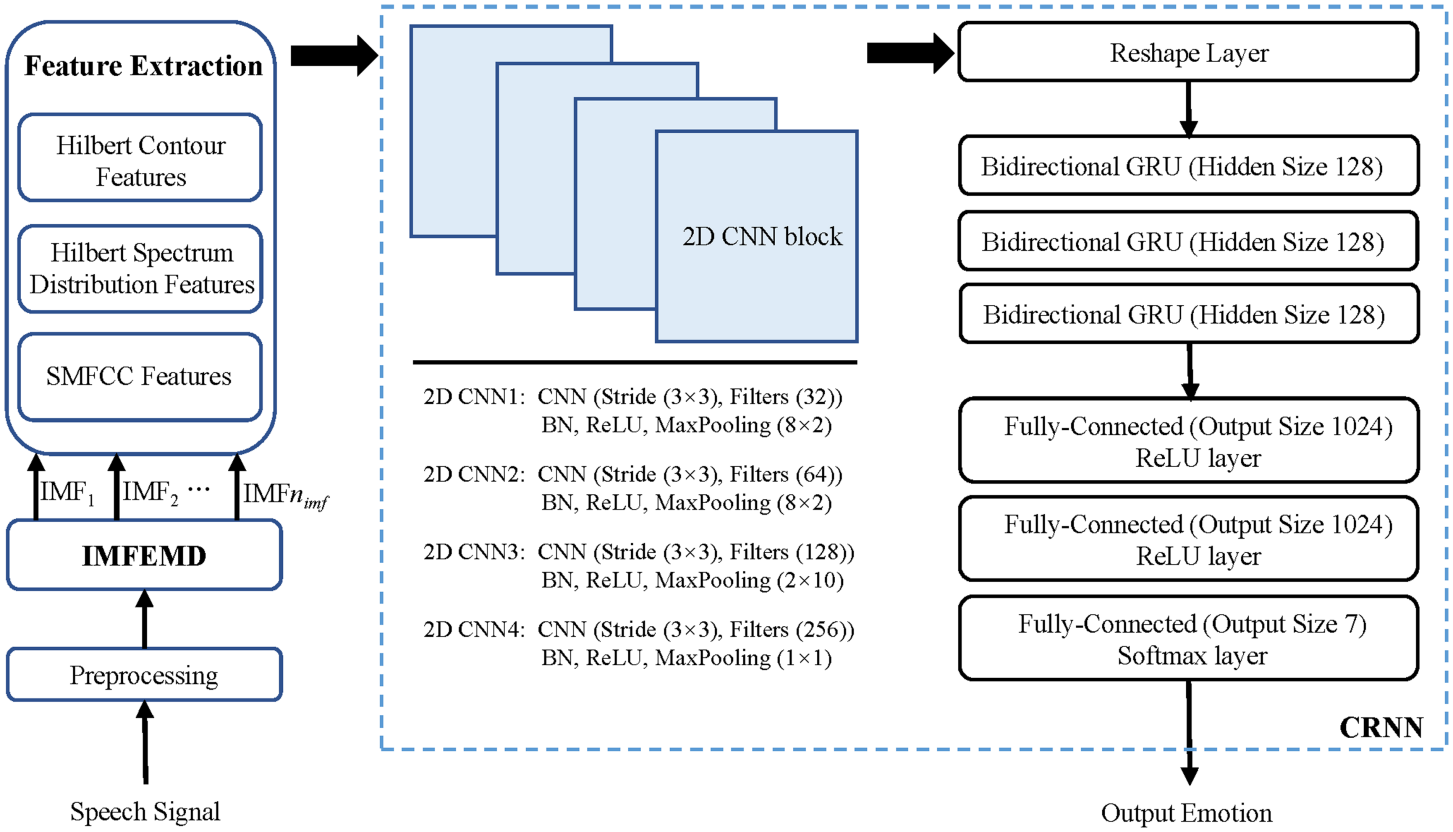
Overall scheme of the IMEMD-CRNN. In the figure, every 2D CNN block (2D CNN) has 4 parts: a 2D CNN layer, a batch normalization layer (BN), a ReLU layer (ReLU), and a 2D max pooling layer (MaxPooling).

In this section, our proposed IMEMD-CRNN to predict emotion is introduced. Figure 1 shows the framework of IMEMD-CRNN. As illustrated, IMEMD-CRNN consists of three modules: IMEMD-based emotional speech signal decomposition, extraction of time-frequency features from IMFs, and speech emotion recognition based on CRNN. Arano et al. (2021) show that effective hand-crafted features, compared to sophisticated deep-learning feature sets, can still have better performance. Therefore, we combine IMEMD-based features with CRNN network in order to improve the robustness and accuracy of the speech emotion recognition system. The framework of IMEMD-CRNN is shown in Figure 1. Design details of the three modules are introduced below.

### 2.1. Improved masking empirical mode decomposition

This part begins with a brief introduction to EMD and MSEMD, and the causes of mode mixing problems are analyzed. Then, we describe our proposed IMEMD.

#### 2.1.1. The masking signal-based EMD

The EMD decomposes a non-stationary signal into a finite and often small number of IMFs and a residue (Huang et al., 1998). The IMFs contain progressively lower frequency components of the signal. The given signal *x*(*t*) can be reconstructed as:


(1) $$\;x\left(t\right)=\sum_{k=1}^{n_{imf}}c_{k}\left(t\right)+r_{es}\left(t\right)$$

where *c_k_*(*t*) (*k* = 1, …, *n_imf_*) represents the *k*th IMF and *r_es_*(*t*) indicates the residue of the signal *x*(*t*). The sifting process of EMD to obtain an IMF from *x*(*t*) is as follows (Huang et al., 1998):

Step 1. Initialize *r*(*t*) = *x*(*t*).
Step 2. Compute all local maxima and minima of *r*(*t*).
Step 3. Interpolating the local maxima (minima) by the cubic spline to obtain the upper (lower) envelope *e_u_*(*t*) (*e_l_*(*t*)) of *r*(*t*).
Step 4. Compute the local mean envelope *e*(*t*) = [*e_u_*(*t*) + *e_l_*(*t*)]/2.
Step 5. Subtract *e*(*t*) from *r*(*t*) and update *r*(*t*) = *r*(*t*) − *e*(*t*).
Step 6. Repeat steps 2 to 5 until *r*(*t*) meets the conditions of IMF.

**Figure 2:**
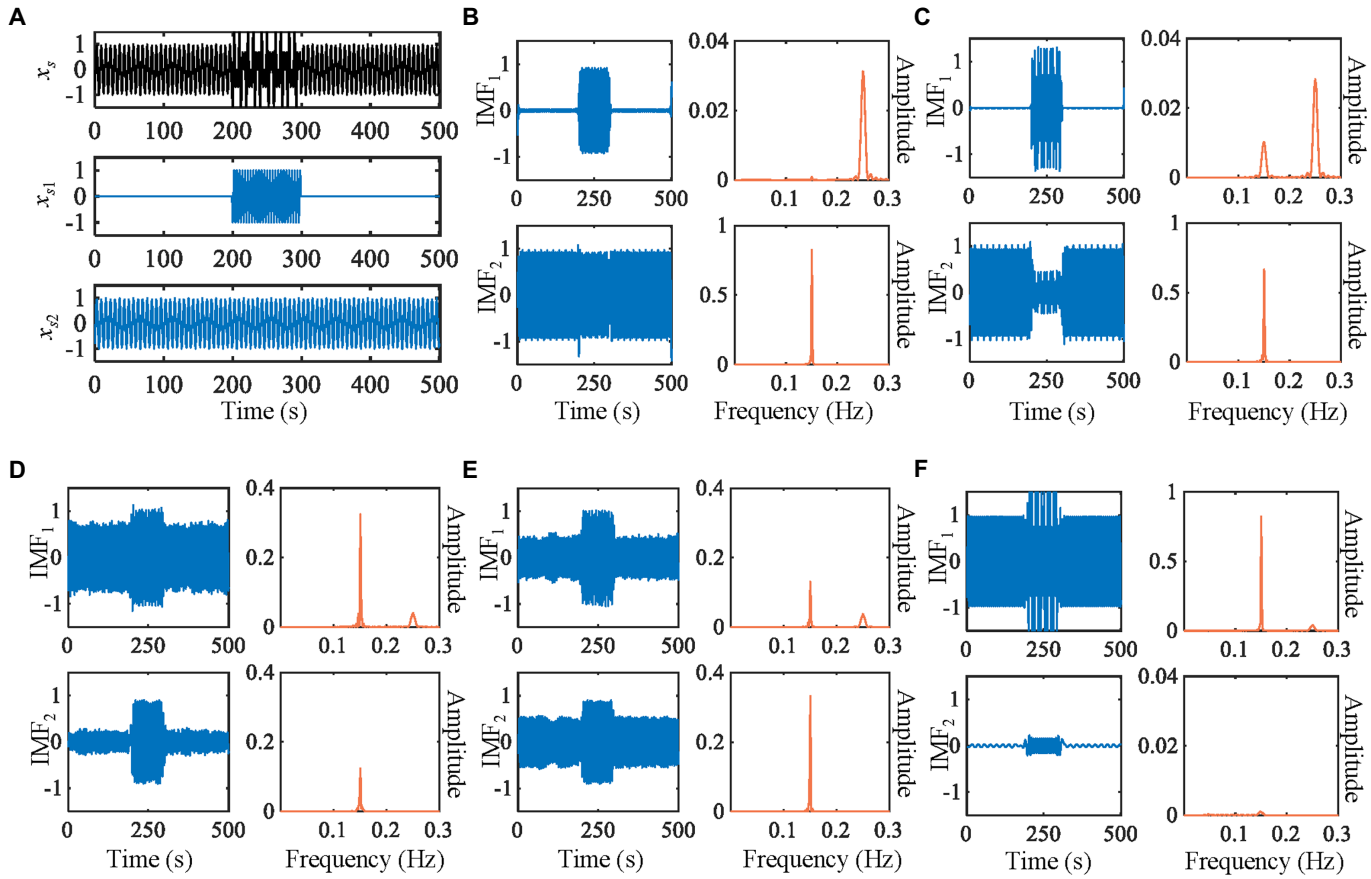
Decomposition of the synthetic signal by five methods. **(A)** The waveforms of synthetic signals. **(B)** IMEMD. **(C)** UPEMD. **(D)** EEMD. **(E)** ICEEMDAN. **(F)** REMD. In each subgraph of **(B–F)**, the left is waveforms of IMFs, the right is power spectra of IMFs.

The mode mixing is that the IMF may contain widely distributed scales (Wu and Huang, 2009). Figures 2C–F show the mode mixing. The mode mixing is mainly caused by the following two situations: (i) intermittency caused by intermittent signal, pulse interference, and noise and (ii) different frequency components of the signal lying within an octave (Deering and Kaiser, 2005; Rilling and Flandrin, 2008). Therefore, many improved algorithms for EMD have been proposed to solve the mode mixing problem. Deering et al. first proposed using masking signals to resolve the mode mixing in EMD (Deering and Kaiser, 2005). The method is called the masking signal-based EMD (MSEMD), which uses a sinusoid signal *x_m_*(*t*) as the masking signal. The process of obtaining an IMF by MSEMD is shown in Algorithm 1 (Shown in Table 1). Let EMD*_k_* (∙) be the operator, which produces the *k*th IMF using EMD. The *β*, *f_w_*, and *θ* represent the amplitude, frequency, and phase of the masking signal, respectively. Their detailed computational process is shown in reference (Deering and Kaiser, 2005). MSEMD has high computational efficiency and can solve mode mixing to some extent, but the parameter selection methods of the masking signal need to be further improved.

**Table 1:** The algorithm to obtain an IMF by MSEMD.

Algorithm 1 Obtaining an IMF by MSEMD	
Function: *c*(*t*) = MSEMD (*x*(*t*))	
Input: *x*(*t*)	
Output: *c*(*t*)	
1:	Construct a masking signal *x_ms_*(*t*) = *β* sin (2π*f_w_t* + *θ*)
2:	Compute *c*_+_(*t*) = EMD_1_(*x*(*t*) + *x_ms_* (*t*))
3:	Compute *c*_−_(*t*) = EMD_1_(*x*(*t*) − *x_ms_* (*t*))
4:	*c*(*t*) = (*c*_+_(*t*) + *c*_−_(*t*))/2

#### 2.1.2. The proposed IMEMD

In this section, we propose a novel method to construct masking signals to alleviate mode mixing. Since our proposed method is an improved version of the MSEMD, it is called improved masking EMD (IMEMD). In IMEMD, obtaining the highest frequency component of the original signal is as follows: First, a masking signal whose frequency is higher than the highest frequency component of the original signal is added to the original signal. Next, the signal is decomposed by EMD, and the first IMF obtained contains the highest frequency component and the masking signal. Then, the masking signal is removed from this IMF to obtain the highest frequency component. The proposed IMEMD is given in Algorithm 2 (Table 2). The value of *ε*_1_ (*ε*_1_ = 30 dB) is referred to as reference (Liu et al., 2017), where *ε*_1_ is the decomposition stop threshold.

**Table 2:** The algorithm of IMEMD.

Algorithm 2 IMEMD	
Function: {*c_k_*(*t*)} = IMEMD (*x*(*t*))	
Input: *x*(*t*)	
Output: {*c_k_*(*t*)}	
1:	Initialize: *n_p_* is the number of phases, *r*_0_(*t*) = *x*(*t*), *k* = 1
2:	While $\int {\left|x\left(t\right)\right|}^{2}dt/\int {\left|r_{k-1}\left(t\right)\right|}^{2}dt<\varepsilon_{1}$ and *r*_*k*-1_(*t*) is not monotonic Do
3:	$c_{k}\left(t\right)=\left(\overset{n_{p}}{\underset{i=1}{\sum }}{\mathrm{EMD}}_{1}\left(r_{k-1}\left(t\right)+v_{ki}\left(t\right)\right)\right)/n_{p}$
4:	*r_k_*(*t*) = *r*_*k-*1_(*t*) − *c_k_*(*t*)
5:	*k* ← *k* + 1
6:	End while
7:	*r_es_*(*t*) = *r*_*k-*1_(*t*)

In Section 2.1.1, we analyze two main reasons for mode mixing: the intermittent components in the signal and the components whose frequencies are within an octave. By adding an appropriate sinusoidal signal (The duration is equal to the original signal) to the original signal, the extrema of the new signal are more uniformly distributed. Thus, the mode mixing due to intermittent components can be alleviated (Wang et al., 2018). At the same time, adding the sinusoidal signal improves the filtering characteristics of the EMD for separating components whose frequencies lie in an octave (Xu et al., 2009). How to construct an appropriate masking signal is shown below:

Our proposed masking signal *v_ki_*(*t*) is represented as follows:


(2) $$v_{ki}\left(t\right)=\xi_{k}\;\sin\left(2\pi \bar{f_{k}}t+2\pi \frac{i-1}{n_{p}}\right)$$

where *ξ_k_* and $\bar{f_{k}}$ are the amplitude and frequency of the *k*th masking signal *v_k_*(*t*), respectively. The parameter *n_p_* is the number of phases (*n_p_*∈*N*, *n_p_* > 1) and *i* = 1, 2, …, *n_p_*.

In the proposed IMEMD, *ξ_k_* and $\bar{f_{k}}$ are determined adaptively according to the nature of the signal, and they are calculated as follows:


(3) $$\xi_{k}=\xi_{0}\cdot \frac{\sum_{t=1}^{T}A_{k}\left(t\right)}{T}$$

(4) $$f_{k}=\frac{\sum_{t=1}^{T}A_{k}\left(t\right)\cdot F_{k}\left(t\right)}{\sum_{t=1}^{T}A_{k}\left(t\right)}$$

(5) $$\bar{f_{k}}=\{\begin{array}{c} f_{k}+f_{k},k=1\\ f_{k-1}+f_{k},k>1 \end{array}$$

where *A_k_*(*t*) and *F_k_*(*t*) are the instantaneous amplitude and frequency of the IMF obtained by EMD_1_(*r*_*k-*1_(*t*)), respectively. *T* is the duration of the signal and $f_{k-1}>f_{k}$ . Following Huang et al. (1998), *A_k_*(*t*) and *F_k_*(*t*) are defined as


(6) $$y_{k}\left(t\right)=\frac{1}{\pi }P\int_{-\infty }^{+\infty }\frac{c_{k}\left(\tau \right)}{\tau -t}d\tau$$

(7) $$A_{k}\left(t\right)=\sqrt{c_{k}^{2}\left(t\right)+y_{k}^{2}\left(t\right)}$$

(8) $$F_{k}\left(t\right)=\frac{1}{2\pi }\cdot \frac{d}{dt}\left(\arctan\frac{y_{k}\left(t\right)}{c_{k}\left(t\right)}\right)$$

where *P* indicates the Cauchy principal value integral, and *y_k_*(*t*) is the Hilbert transform (HT) of the *k*th IMF, *c_k_*(*t*).

Equations 2–8 describe the calculation of the frequency, amplitude, and phase of the mask signal in the proposed IMEMD. For the masking frequency, studies have shown that two components with a frequency ratio between 0.5 and 2 can be separated when the frequency of the mask signal is higher than the frequency of the high-frequency component (Senroy et al., 2007; Rilling and Flandrin, 2008). For signal *x*(*t*), when its two adjacent frequency components *f*_tr,*k*_ and *f*_tr,*k* + 1_ satisfy


(9) $$1<\frac{f_{tr,k}}{f_{tr,k+1}}<2$$

and the mode mixing occurs after the EMD_1_(*x*(*t*)) operation, $\;f_{k}>f_{tr,k+1}\;\left(k=1\right)$ , hence $f_{tr,k}<2\;f_{k}$ . When *k* > 1 and the mode mixing occurs after the EMD_1_(*r*_*k-*1_(*t*)) operation, $\;f_{k-1}>f_{tr,k}$ (*k* > 1), hence $f_{tr,k}<f_{k}+f_{k-1}$ . So, the masking frequency $\bar{f_{k}}$ in Equation 5 still satisfies that the frequency of the mask signal is higher than the frequency of the high-frequency component. Wang et al. (2018) prove that the residual noise can be reduced by using a few sinusoidal signals with uniform phase distribution as masking signals. Therefore, in obtaining the *k*th IMF by IMEMD, we construct *n_p_* mask signals whose phases are uniformly distributed over the 2π space. Then, the new signals after adding these *n_p_* mask signals are decomposed by EMD, respectively, to obtain *n_p_* IMFs. The mean of these *n_p_* IMFs is used as the final *k*th IMF, which can reduce the residual of the mask signals in the decomposition results and decrease the decomposition error. The effect of the different number of phases on the signal reconstruction error is experimentally analyzed in Section 3.3.1. In the power quality detection task, the appropriate masking amplitude can be determined based on the amplitude of the frequency component obtained by fast Fourier transform (FFT) (Wu et al., 2014). Inspired by this, we use instantaneous amplitudes obtained based on the HT to construct masking amplitude. Since the HT-based instantaneous amplitudes are time-varying, we average all instantaneous amplitudes during *T*. In Equations 2, 3, the values of *n_p_* (*n_p_* = 64) and $\xi_{0}$ ( $\xi_{0}$ = 1.5) are empirical. In Section 3.3, we test the effect of different values of *n_p_* and $\xi_{0}$ on the IMF estimation.

### 2.2. Feature extraction based on IMEMD

In this section, we extract two feature sets for SER using IMEMD. The first feature set is the timbre features proposed in our previous work (Li et al., 2020b). Timbre features are proven to be essential features for SER (Guidi et al., 2019). The other feature set is the Mel-frequency cepstral coefficients based on the reconstructed signal (SMFCC), which has been proven effective in distinguishing different speech emotions (Kerkeni et al., 2019). The following are details of two feature sets used in IMEMD-CRNN. Table 3 shows the details of these two feature sets.

**Table 3:** The feature sets extracted by IMEMD for SER.

Category	Feature name	Dimensions
Timbre features	Hilbert spectrum distribution features (*SC*, *SP*, *SK*, *SU*)	4
	Hilbert contour features (*SE*, ∆*SE*, ∆^2^*SE*)	3
Spectral features	SMFCC	12
	First derivative of SMFCC (∆*SMFCC*)	12
	Second derivative of SMFCC (∆^2^*SMFCC*)	12

#### 2.2.1. Timbre features based on IMEMD

IMEMD method is first adopted in this section to extract the intrinsic mode functions of speech. Then, the timbre feature sets, including the Hilbert spectrum distribution features and Hilbert contour features, are extracted.

For each frame of the signal, Hilbert spectrum distribution features are calculated as follows


(10) $$\;SC=\frac{\sum_{k=1}^{n_{imf}}F_{ce}\left[k\right]\cdot E_{me}\left[k\right]}{\sum_{k=1}^{n_{imf}}E_{me}\left[k\right]}$$

(11) $$\;SP=\sqrt{\frac{\sum_{k=1}^{n_{imf}}E_{me}\left[k\right]\cdot {\left(F_{ce}\left[k\right]-SC\right)}^{2}}{\sum_{k=1}^{n_{imf}}E_{me}\left[k\right]}}$$

(12) $$\;SK=\frac{\sum_{k=1}^{n_{imf}}E_{me}\left[k\right]\cdot {\left(F_{ce}\left[k\right]-SC\right)}^{3}}{SP^{3}\sum_{k=1}^{n_{imf}}E_{me}\left[k\right]}$$

(13) $$\;SU=\frac{\sum_{k=1}^{n_{imf}}E_{me}\left[k\right]\cdot {\left(F_{ce}\left[k\right]-SC\right)}^{4}}{SP^{4}\sum_{k=1}^{n_{imf}}E_{me}\left[k\right]}$$

where *F_ce_*[*k*] is the centroid frequency calculated for the instantaneous frequency of one frame in the *k*th IMF. *E_me_*[*k*] is the mean value of the instantaneous amplitude of one frame in the *k*th IMF.

For each frame of the signal, Hilbert contour features are calculated as follows:


(14) $$\;SE=\max\left(E_{me}\left[k\right]\right)$$

(15) $$\Delta SE\left(\varphi \right)=\{\begin{array}{c} SE\left(\varphi +1\right)-SE\left(\varphi \right),1\leq \varphi \leq Q\\ \frac{\sum_{q=1}^{Q}q\left(SE\left(\varphi +q\right)-SE\left(\varphi -q\right)\right)}{\sqrt{2\sum_{q=1}^{Q}q^{2}}},Q<\varphi \leq \Phi -Q\\ SE\left(\varphi \right)-SE\left(\varphi -1\right),\Phi -Q<\varphi \leq \Phi \end{array}$$

where Φ is the total number of frames of the signal. The second derivative ∆^2^*SE* can be solved by replacing the *SE* in the above equation with ∆*SE* where *Q* is the time difference of the first derivative, which is usually taken as 2.

#### 2.2.2. Spectral features based on IMEMD

We extract the Mel-frequency cepstral coefficients based on the reconstructed signal (*SMFCC*) (Kerkeni et al., 2019) as the features to characterize speech emotions. The reconstructed signal is obtained by IMEMD. In order to improve the accuracy of speech emotion recognition, we also extract the first derivative of *SMFCC* (∆*SMFCC*) and the second derivative of *SMFCC* (∆^2^*SMFCC*). Because derivative features contain some temporal information, research show that this information is essential for speech emotion recognition (Kerkeni et al., 2019).

First, we use the zero-crossing rate detection method to find the signal trend *x_tr_*(*t*), as shown in Equation 16.


(16) $$x_{tr}=\underset{k}{\sum }c_{k}\left(t\right),\mathrm{if}\;\frac{ZeroCross_{c_{k}\left(t\right)}}{ZeroCross_{c_{1}\left(t\right)}}\;\left(k=2,3,..,n_{imf}\right)$$

where $ZeroCross_{c_{k}\left(t\right)}$ is the zero-crossing rate. Then, *x_tr_*(*t*) is subtracted from the original signal, and the rest of the signal is used to reconstruct the original signal. The *SMFCC* is obtained by calculating the MFCCs with 12 orders of the reconstructed signal. Thus, for the reconstructed signal, the number of *SMFCC* coefficients returned per frame is 12; that is, the dimension of *SMFCC* features is 12.

The ∆*SMFCC* and ∆^2^*SMFCC* describe the trajectories of *SMFCC* over time. When the number of frames of the reconstructed signal is Φ , the first derivative of φ th frame ΔSMFCC(φ) is calculated as follows:


(17) $$\Delta SMFCC\left(\varphi \right)=\{\begin{array}{c} SMFCC\left(\varphi +1\right)-SMFCC\left(\varphi \right),1\leq \varphi \leq Q\\ \frac{\sum_{q=1}^{Q}q\left(\begin{array}{l} SMFCC\left(\varphi +q\right)\\ -SMFCC\left(\varphi -q\right) \end{array}\right)}{\sqrt{2\sum_{q=1}^{Q}q^{2}}},Q<\varphi \leq \Phi -Q\\ SMFCC\left(\varphi \right)-SMFCC\left(\varphi -1\right),\Phi -Q<\varphi \leq \Phi \end{array}$$

where *Q* is the time difference of the first derivative, which is usually taken as 2. The second derivative is calculated in the same way, but it is calculated from ΔSMFCC(φ) , not *SMFCC*. Thus, the number of dimensions of ∆*SMFCC* and ∆^2^*SMFCC* features is also 12.

### 2.3. Convolutional recurrent neural network

The architecture of CRNN in this paper is based on Adavanne et al. (2019) and Cao et al. (2019). The CRNN contains three parts. The first part includes four 2D CNN blocks and a reshape layer. Each of these 2D CNN blocks consists of a batch normalization layer (BN), a ReLU layer (ReLU), and a 2D max pooling layer (MaxPooling). The second part has three bidirectional GRUs. The third part has three fully connected layers. The output layer uses the softmax activation function. The cross-entropy loss is used to train the network and is optimized using an Adam optimizer. We train the network for 60 epochs with a mini-batch size of 512. The initial learning rate $\eta_{0}$ is 0.001. The architectural details of CRNN are shown in Figure 1.

## 3. Results and discussion

### 3.1. Datasets

#### 3.1.1. Synthetic signals

The synthetic signals to evaluate the performance of our IMEMD is a classical mode mixing example (shown in Figure 2). The synthetic signal *x_s_*(*t*) consists of a sustained pure tone *x*_*s*1_(*t*) and a gapped one *x*_*s*2_(*t*) with a higher frequency, where their frequencies lie within an octave. The data *x_s_*(*t*) = *x*_*s*1_(*t*) + *x*_*s*2_(*t*) is sampled at 1 Hz rate, 0≤t≤500 , with


(18) $$x_{s1}\left(t\right)=\{\begin{array}{c} \sin\left(2\pi \cdot 0.25\cdot \left(t-201\right)\right),201\leq t\leq 300\\ 0,t<201\;or\;t>300 \end{array}$$

(19) $$x_{s2}\left(t\right)=\sin\left(2\pi \cdot 0.15\cdot \left(t-1\right)\right),0\leq t\leq 500$$

#### 3.1.2. Public datasets

The IMEMD-CRNN system is validated on the Berlin Emotional Database (Emo-DB; Burkhardt et al., 2005) and Toronto Emotional Speech Set (TESS; Pichora-Fuller and Dupuis, 2020). They are the most popularly used databases for emotion recognition (Deb and Dandapat, 2019). Both datasets were approved by ethical committees. The Emo-DB dataset includes 535 audio files simulated by 10 actors on 10 German utterances. All files are in 16-bit stereo wave sampled at 16 kHz and labeled with one of the 7 emotions. The average duration of the utterances in this dataset is 3.5 s, and the approximate duration of the utterances is 3 s to 5 s. The number of emotional labels across the dataset is anger (127), anxiety/fear (69), boredom (81), disgust (46), happiness (71), neutral (79), and sadness (62). Audio files in the Emo-DB are single-channel audio.

The TESS database is recorded by two actresses aged 26 and 64. Both actresses speak English as their first language. There are 2,800 audio samples in the database, including seven different emotions: anger, disgust, fear, happiness, pleasant surprise, sadness, and neutral. There are 400 data samples for each emotion. The sampling rate is 24.414 kHz and is saved in WAV format with all audio samples between 2 s and 3 s in length. Audio files in the TESS are single-channel audio.

### 3.2. Preprocessing and evaluation metrics

Utterances in TESS and Emo-DB datasets are recorded in a noise-less environment; therefore, there is no need to filter and denoise the data (Krishnan et al., 2021). Utterances in the two datasets are split into equal-length segments of 3 s, and zero padding is used for utterances with a duration of less than 3 s (Chen et al., 2018). Each utterance is normalized by dividing the time-domain signal by its maximum value. For each utterance (sampling rate: 16 kHz for Emo-DB, 24.414 kHz for TESS), the frame size is uniformly set to 25 ms, and the hop size is 10 ms. To improve the performance of our IMEMD-CRNN architecture, we use data augmentation techniques to enlarge the size of the Emo-DB dataset, and every file is enlarged to 60 augmentations. We enlarge the Emo-DB dataset with three data enhancement methods: pitch shifting, time shifting, and noise addition. For pitch shifting, the range of pitch shift in semitones is [−2, 2]. The range of time shift in seconds is [−0.4, 0.4]. We use the Gaussian white noise addition, and the range of noise SNR in dB is [−20, 40]. Each audio is normalized by dividing the time-domain signal by its maximum value.

When evaluating our proposed IMEMD, the reconstruction error of the reconstructed signal $\tilde{x}$ relative to the original signal *x* is measured by the relative root mean square error (RRMSE), and the calculation formula is as follows:


(20) $${\mathrm{RRMSE}}_{x}\left(\tilde{x}\;\right)=\frac{\sqrt{\sum_{n=1}^{N}{\left({\tilde{x}}_{n}-x_{n}\right)}^{2}}}{\sqrt{\sum_{n=1}^{N}x_{n}{}^{2}}}$$

To compare with the state-of-the-art SER methods, we use unweighted accuracy (UA) to evaluate the performance of different SER methods (Zhong et al., 2020).

### 3.3. Performance of IMEMD

#### 3.3.1. Simulations and comparisons

**Figure 3:**
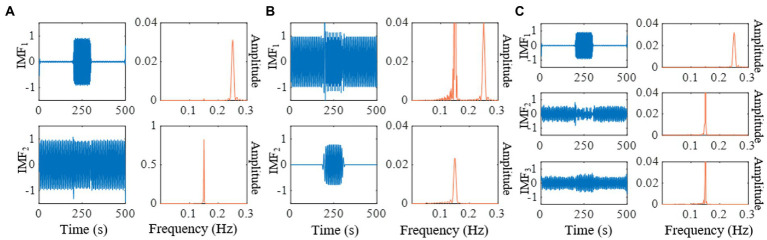
Decomposition of the synthetic signal by IMEMD. **(A)** The $\xi_{0}$ =1.5. **(B)** The $\xi_{0}$ =0.1. **(C)** The $\xi_{0}$ =3.

We compare the results of IMEMD with those of EEMD, UPEMD, ICEEMDAN, and REMD in Figure 2 through the decomposition of the artificial signal. We only show the first two IMFs of these methods as the mode mixing mainly occurs in the first two modes of the artificial signal. We set the noise standard deviation to 0.4, the ensemble size to 100, and phase number to 16 for EEMD, UPEMD, and ICEEMDAN, which are similar to those in Colominas et al. (2014) and Wang et al. (2018). For IMEMD, we set *n_p_* = 64 and *ξ*_0_ = 1.5 through experiments. The number of IMF obtained by IMEMD, REMD, UPEMD, ICEEMDAN, EEMD, and EMD is 2, 3, 8, 12, 14, and 14, respectively. In Figure 2, when separating components whose frequencies lie within an octave, the separation degree of each method from high to low is IMEMD > UPEMD > ICEEMDAN > EEMD > REMD> EMD. IMEMD substantially reduces the mode mixing. The proper value of *ξ*_0_ greatly impacts the performance of IMEMD and in this work, *ξ*_0_ is empirical. In Figure 3, three case studies are performed to show the effect of *ξ*_0_ on mode estimation by IMEMD. The values of other parameters are the same as in Figure 2. Figures 3A–C show the decomposition of the synthetic signal by IMEMD when *ξ*_0_ is taken as the most appropriate value, *ξ*_0_ increase to a large value, and *ξ*_0_ increase to a small value, respectively. As shown in Figure 3B, when the value of *ξ*_0_ is too small, there are *x*_*s*1_(*t*) and *x*_*s*2_(*t*) in IMF1. In Figure 3C, when the value of *ξ*_0_ is too large, *x*_*s*2_(*t*) appears in IMF2 and IMF3. The results in Figures 3B,C are mode mixing. These mean that inappropriate values of *ξ*_0_ can cause mode mixing problems.

**Figure 4:**
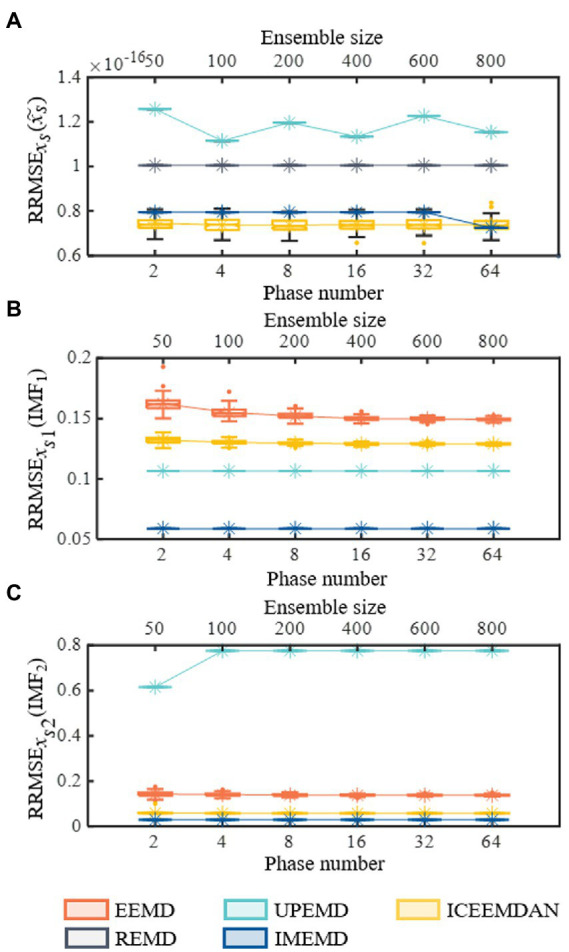
Performances of recovering known components on synthetic signal *x_s_*(*t*). All five methods are decomposed 100 times to obtain the statistical average results and shown using boxplots. **(A)** Reconstruction errors of synthetic signal *x_s_*(*t*). **(B)** Performances of recovering *x*_*s*1_(*t*). **(C)** Performances of recovering *x*_*s*2_(*t*). In each subgraph of **(A–C)**, the symbol “*” represents the mean value of the corresponding 100 decomposition results.

In order to better compare the reconstruction errors of different methods in a different number of trials (the results are shown in Figure 4), we set the frequency of *x*_*s*2_(*t*) to 0.07. So, frequencies of *x*_*s*1_(*t*) and *x*_*s*2_(*t*) do not lie within an octave. Assisted signals with an amplitude of 0.2 are utilized for EEMD, ICEEMD, and UPEMD (Wang et al., 2018). Ensemble sizes of EEMD and ICEEMDAN are set to *I* = 50, 100, 200, 400, 600, and 800 (Wu and Huang, 2009; Colominas et al., 2014). Masking signals with phase numbers *n_p_* = 2, 4, 8, 16, 32, and 64 are used in UPEMD and IMPEMD (Wang et al., 2018) to explore the effect of phase numbers on the decomposition results of the algorithms. Moreover, 10 sifting iterations are used to extract IMFs for all methods. In order to quantify the performance of the methods, all methods are decomposed 100 times to obtain the statistical average results (shown in Figure 4). Figure 4 shows that when *n_p_* > 32, reconstruction errors (the value is 7.25 × 10^−17^) of *x_s_*(*t*) by IMEMD are smaller than those of ICEEMDAN (the value is 7.38 × 10^−17^). For all values of *n_p_*, reconstruction errors of *x_s_*(*t*) by IMEMD are about one-tenth of the reconstruction errors of *x_s_*(*t*) by UPEMD. When the number of phases *n_p_* ranges from 2 to 64, the reconstruction errors of *x*_*s*1_(*t*) and *x*_*s*2_(*t*) reconstructed by IMEMD have little changes, and the reconstruction errors of *x_s_*(*t*) reconstructed by IMEMD decrease. When *n_p_* = 64, the reconstruction errors of these signals decomposed by IMEMD are small enough and smaller than these of the compared algorithms. Moreover, the time complexity of IMEMD is increasing as *n_p_* increases, so we set the value of *n_p_* in the IMEMD to 64. Reconstruction errors of *x_s_*(*t*) using EEMD are greater than 0.07. This may be because the signal contains a lot of residual noise. Therefore, the results of EEMD are not drawn in Figure 4A. Figures 4B,C plot errors of recovering *x*_*s*1_(*t*) and *x*_*s*2_(*t*), respectively. As shown in Figure 4, IMEMD is better than the other methods. The reconstruction error of *x*_*s*1_(*t*) and *x*_*s*2_(*t*) obtained by REMD is the largest among all the compared algorithms. For *x*_*s*1_(*t*), the reconstruction error obtained by REMD is more than 12 times higher than that of EEMD, which has the second-highest reconstruction error. For *x*_*s*2_(*t*), the reconstruction error obtained by REMD is more than 1.2 times higher than that of UPEMD, which has the second-highest reconstruction error. Therefore, the results of REMD are not drawn in Figures 4B, 3C. The boxplots in Figure 4 show that the distribution of results obtained by IMEMD and UPEMD is more concentrated than that obtained by ICEEMDAN and EEMD. This is because perturbations used by IMPEMD and UPEMD are deterministic, while ICEEMDAN and EMD use random noise. So IMEMD and UPEMD can obtain reproducible decompositions. In conclusion, the IMEMD proposed in this paper reduces the mode mixing effect, provides reproducible decompositions, and has less computational time.

#### 3.3.2. Emotional speech and comparisons

**Figure 5:**
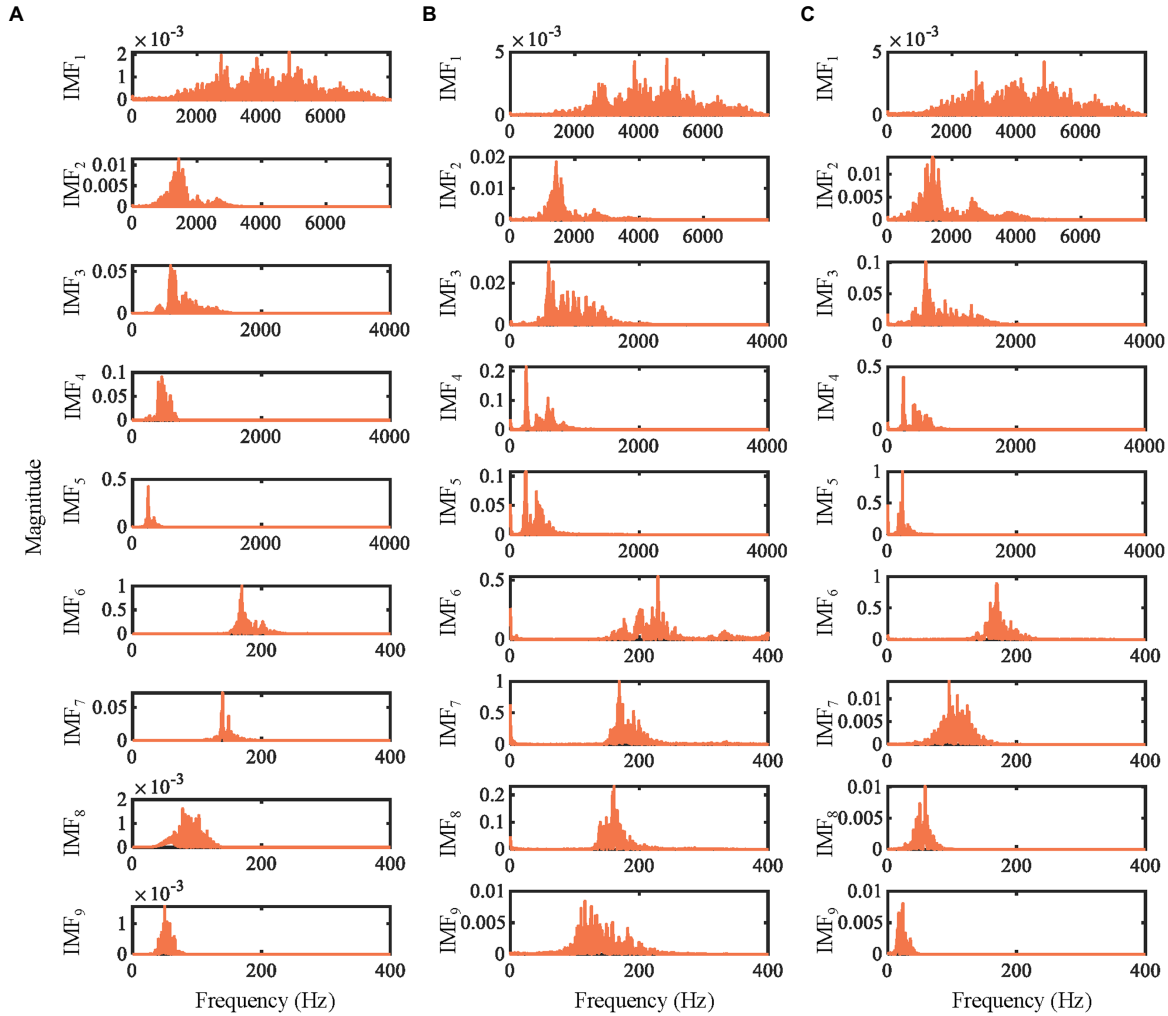
The power spectra of the first 9 IMFs obtained by decomposing the emotional speech signal by different methods. **(A)** IMEMD. **(B)** ICEEMDAN. **(C)** UPEMD.

IMEMD is applied to real emotional speech (from the Emo-DB dataset) shown in Figure 5. Figure 5 shows the power spectra of the first 9 IMFs. The spectra of IMFs by each algorithm are normalized by dividing the spectra by their maximum magnitudes. As shown in Section 3.3.1, the reconstruction errors of EEMD and REMD are large. Therefore, IMEMD is only compared with UPEMD and ICEEMDAN. The phase number of *n_p_* = 64 is used in IMEMD and UPEMD. The ensemble size of ICEEMDAN is *I* = 100. We set *ξ*_0_ = 1.5 for IMEMD and the amplitude of assisted signals to 0.2 for UPEMD and ICEEMDAN.

In Figure 5, the mode mixing of IMEMD is less than that of other methods. For ICEEMDAN and UPEMD, there is mode mixing between IMF2 and IMF3, and between IMF4 and IMF5. The number of IMFs obtained by IMEMD, UPEMD, and ICEEMDAN is 14, 15, and 23, respectively, which proves that IMEMD can return a more compact representation than other methods. Noise residuals and mode mixing effects have bad effects on the frequency distribution of the IMFs, resulting in the spectrum becoming blurry (Sandoval and De Leon, 2017). So, the performance of IMEMD is better than that of UPEMD and ICEEMDAN.

### 3.4. Performance analysis of IMEMD-CRNN system

In this section, the proposed IMEMD-CRNN method is applied to the two publicly available Emo-DB and TESS datasets for speech emotion recognition experiments to show the significance and the robustness of the IMEMD-CRNN method. In the upcoming subsections, the experimental results will be described in detail.

#### 3.4.1. Performance on the Emo-DB dataset

The utterances on the Emo-DB dataset are spoken by 10 actors intended to convey one of seven emotions. These seven emotion labels are anger, anxiety/fear, boredom, disgust, happiness, neutral, and sadness. We first preprocess each utterance (The preprocessing method is shown in Section 3.2). Secondly, the signal is decomposed by IMEMD to obtain IMFs. Then, we extract Hilbert spectrum distribution features, Hilbert contour features, SMFCC features, the first derivative of SMFCC, and the second derivative of SMFCC from IMFs (The feature extraction method is shown in Section 2). The dimension of features is 43. We use leave-one-speaker-out (LOSO) 10-fold cross-validation to provide an accurate assessment of the proposed IMEMD-CRNN model (Hou et al., 2022). In the LOSO 10-fold cross-validation method, utterances of 8 speakers are used as training set, one speaker is selected as the validation data, and utterances of the left-out speaker are used as the testing set. We repeat this procedure 10 times. The final classification accuracy is the average of the 10 folds. The initial values of hyperparameters of the CRNN model are referred to Adavanne et al. (2019) and Cao et al. (2019). We further utilize the validation set to debug the hyperparameters to obtain optimal hyperparameters.

Table 4 shows the recognition results of the proposed method with state-of-the-art (SOTA) methods. The unweighted accuracy of our method reaches 93.54%, greater than the SOTA method by 1.03%. To verify that the improvement in accuracy of the proposed method is statistically significant compared to the SOTA method (the method proposed by Hou et al. (2022)), a paired-sample *t*-test is used. The null hypothesis is that the pairwise difference between the UA of the two methods has a mean equal to zero. The significance level *α* of the hypothesis test is set to 0.05. The *value of p* of the paired-sample *t*-test is 0.01 (*p* < 0.05). Therefore, the improvement in the accuracy of IMEMD-CRNN compared with SOTA method is statistically significant. As shown in Table 4, combining hand-crafted features with deep learning is higher than the methods where the original signals are directly fed into the deep networks. The results demonstrate that effective hand-crafted features combined with deep-learning networks can build a more accurate and robust speech emotion recognition system. The accuracies obtained using our method for each emotion are anger (90.9%), anxiety/fear (96%), boredom (92.4%), disgust (97.6%), happiness (90%), neutral (92.8%), and sadness (95.1%). The results indicate that our proposed IMEMD-CRNN framework has the best performance for disgust and the worst performance for anger and happiness. Some angry samples are identified as happiness and anxiety. A part of happy samples is recognized as angry and anxious. This may be because all three emotions are relatively strong and, therefore, easily misclassified.

**Table 4:** Comparison of different SER methods on the EMO-DB dataset.

Methods	Input feature	UA (%)
Deb and Dandapat (2019)	MFCCs and their first- and second-order difference	85.10
Suganya and Charles (2019)	Raw audio recording	85.62
Kerkeni et al. (2019)	Modulation spectral and modulation frequency features based on EMD and TKEO, and cepstral features.	86.22
Chen et al. (2018)	Log Mel-spectrogram	87.81
Muppidi and Radfar (2021)	RGB Mel-spectrogram	88.78
Kim et al. (2018)	20 features in the eGeMAPS	88.90
Mustaqeem and Kwon (2021)	Raw audio recording	89.37
Zhong et al. (2020)	Log Mel-spectrogram	90.67
Hou et al. (2022)	Prosody features, MFCCs, MFSCs	92.51
**Proposed**	**Timbre features, spectral features**	**93.54**

#### 3.4.2. Performance on the TESS dataset

To compare with other SER methods, we use randomized 10-fold cross-validation to train and validate our method on the TESS dataset. The final performance is the averaged results of the 10 folds. The preprocessing and feature extraction steps are the same as the Emo-DB database. The initial values of hyperparameters of the CRNN model are referred to Adavanne et al. (2019) and Cao et al. (2019). We further utilize the validation set to debug the hyperparameters to obtain optimal hyperparameters. Table 5 shows the results of comparing the proposed method with the state-of-the-art method on the TESS dataset. From Table 5, it can be seen that the proposed method achieves a UA value of 100% in the TESS database; the UA value is improved by 4.21% compared to the best comparison method. We also use the paired-sample *t*-test to compare the results of IMEMD-CRNN and the method proposed by Chatterjee et al. (2021). The significance level *α* of the hypothesis test is set to 0.05. The value of *p* of the paired-sample *t*-test is 6.24 × 10^−7^ (*p* < 0.05). Therefore, the improvement in the accuracy of IMEMD-CRNN compared with the SOTA method is statistically significant.

**Table 5:** Comparison of different SER methods on the TESS dataset.

Methods	Input feature + Classifier	UA (%)
Krishnan et al. (2021)	Entropy features based on EMD + SVM	81.67
Krishnan et al. (2021)	Entropy features based on EMD + LDA	93.30
Chatterjee et al. (2021)	MFCCs +1D CNN	95.79
**Proposed**	**Timbre and spectral features + CRNN**	**100**

## 4. Conclusion

This paper proposes a novel framework named IMEMD-CRNN to accurately extract emotional information from speech and effectively identify different emotions. The IMEMD-CRNN contains three parts. IMEMD is first used to extract physically meaningful IMFs from speech signals. Then, we extracted time-frequency features from the IMFs that can effectively express speech emotions. Finally, CRNN is employed to further model the speech emotion information in the time-frequency features to realize the recognition of emotion. Comprehensive experiments on the synthetic signals, the Emo-DB dataset, and TESS dataset verify the effectiveness of the proposed scheme. Simultaneously, simulations and emotional speech experiments indicate that our IMEMD mitigates mode mixing and improves decomposition accuracy under low computational cost. More importantly, we compare our proposed scheme with some state-of-the-art SER methods. The results show that our method can accurately extract speech emotion features and significantly improves the performance of SER. The proposed IMEMD-CRNN framework has potential applications in psychology, physiology, signal processing, and pattern recognition involving speech-based affective computing. In future work, to further reduce the mode mixing and improve the ability of IMEMD to decompose signals, the addition of optimization algorithms to the IMEMD will be investigated.

## Data availability statement

The original contributions presented in the study are included in the article/supplementary material, further inquiries can be directed to the corresponding author.

## Author contributions

CS was involved in experiment conduction, data analysis, and manuscript write-up. HL and LM were involved in the conception, supervision, and manuscript review. All authors contributed to the article and approved the submitted version.

## Funding

This work was supported in part by the National Natural Science Foundation of China under Grant U20A20383, in part by the National Key R&D Program of China under Grant 2020YFC0833204, Provincial Key R&D Program of Heilongjiang under Grant GY2021ZB0206, Shenzhen Foundational Research Funding under Grant JCYJ20200109150814370, and Funds for National Scientific and Technological Development under Grant 2021SZVUP087 and Grant 2021SZVUP088.

## Conflict of interest

The authors declare that the research was conducted in the absence of any commercial or financial relationships that could be construed as a potential conflict of interest.

## Publisher’s note

All claims expressed in this article are solely those of the authors and do not necessarily represent those of their affiliated organizations, or those of the publisher, the editors and the reviewers. Any product that may be evaluated in this article, or claim that may be made by its manufacturer, is not guaranteed or endorsed by the publisher.
